# Metabolic Rewiring of GPC3-Targeted CAR T Cells Expressing IL-15 and HER2-Targeted CAR T Cells via the ADA1–CD26 Axis Promotes Tumor Infiltration and Limits Systemic Toxicities

**DOI:** 10.3390/biomedicines14102258

**Published:** 2026-10-06

**Authors:** Yue Hu, Kevin Song, Ruoning Wang, Stephen Gottschalk, Andras Heczey, Xiaotong Song

**Affiliations:** 1 Center for Inflammatory and Infectious Diseases, Institute of Biosciences and Technology, Texas A&M University, Houston, TX 77030, USA; 2 Department of Translational Medical Sciences, Texas A&M University, Houston, TX 77030, USA; 3 Department of Biology, University of Houston, Houston, TX 77004, USA; 4 Center for Childhood Cancer Research, Nationwide Children’s Hospital, Columbus, OH 43205, USA; 5 Hematology, Oncology & Blodd and Marrow Transplant, Abigail Wexner Research Institute, Nationwide Children’s Hospital, Columbus, OH 43205, USA; 6 Department of Pediatrics, The Ohio State University, Columbus, OH 43205, USA; 7 Department of Bone Marrow Transplantation & Cellular Therapy, St. Jude Children’s Research Hospital, Memphis, TN 38105, USA; 8 Hematology-Oncology, Seattle Children’s Hospital, University of Washington School of Medicine, Seattle, WA 98105, USA

**Keywords:** CAR T cell, IL-15, ADA1, CD26, solid tumor, persistence, invasion, cytokine release syndrome, inflammation, memory T cells, macrophage polarization

## Abstract

**Background:** Chimeric antigen receptor (CAR) T-cell therapy for solid tumors is limited by inadequate T-cell persistence and tumor infiltration, as well as treatment-associated cytokine release syndrome (CRS). To address these challenges, we developed a metabolically rewired CAR T-cell (MRCAR) platform by co-expressing adenosine deaminase 1 (ADA1) and CD26 in CAR T cells. **Methods:** MRCAR T cells were generated using either GPC3-targeted CARs for hepatocellular carcinoma (HCC) or HER2-targeted CARs for breast cancer. For HCC, we further evaluated constructs with and without enforced IL-15 expression to boost T-cell function. **Results:** In vitro, IL-15–expressing GPC3-MRCAR T cells exhibited enhanced proliferation, sustained effector function, reduced exhaustion, and superior cytotoxicity following repeated antigen stimulation compared with conventional CAR T cells. CD26 expression significantly enhanced CAR T-cell invasiveness in transwell assays and promoted tumor infiltration in vivo in both tumor models. ADA1 expression enhanced CAR T-cell persistence and was associated with increased frequencies of memory and stem-like T-cell populations in vivo. Notably, although IL15-GPC3-CAR T-cell treatment increased antitumor activity, it also caused high mortality and systemic inflammation in Huh7 tumor-bearing mice, whereas incorporation of ADA1 and CD26 into MRCAR T cells significantly improved survival and reduced serum levels of IL-6, IFN-γ, and TNF-α. Mechanistically, conventional IL-15–expressing GPC3-CAR T cells induced proinflammatory M1-like macrophage activation, whereas MRCAR T cells markedly suppressed this inflammatory response. **Conclusions:** Collectively, these results indicate that metabolic rewiring via ADA1 and CD26 enhances CAR T-cell expansion, tumor infiltration, persistence, and safety, supporting the MRCAR platform as a promising strategy to improve both efficacy and tolerability of CAR T-cell therapy for solid tumors.

## 1. Introduction

Adoptive cell therapies employing chimeric antigen receptor (CAR) T cells have transformed the treatment landscape for hematologic malignancies, providing unprecedented rates of durable remissions in diseases such as acute lymphoblastic leukemia and diffuse large B-cell lymphoma [1,2,3,4,5,6,7]. Despite these successes, CAR T cell therapy in solid tumors remains largely ineffective due to major barriers, including insufficient T cell persistence, limited tumor infiltration, rapid functional exhaustion, and the risk of severe cytokine release syndrome (CRS) [8,9]. The immunosuppressive microenvironment of solid tumors, characterized by poor nutrient availability, high concentrations of inhibitory metabolites such as adenosine, and the presence of immunosuppressive myeloid cells, greatly impedes CAR T cell functionality [10,11,12,13,14,15,16,17,18].

To overcome these obstacles, recent efforts have focused on modifying CAR T cell metabolism, differentiation, and migratory capacity. Strategies to improve T cell persistence, such as engineering cytokine signaling (e.g., IL-15 expression), enhancing memory phenotypes, and increasing resistance to exhaustion, have yielded incremental improvements [19,20]. However, potent CAR T cell expansion often comes at the cost of increased risk for CRS, driven by excessive pro-inflammatory cytokine release [20]. Thus, achieving enhanced anti-tumor efficacy without escalating toxicity remains a primary goal in the clinical translation of CAR T cell therapy for solid tumors.

Adenosine deaminase 1 (ADA1) is a central enzyme in purine metabolism that catalyzes the conversion of adenosine, an immunosuppressive metabolite abundant in solid tumors, into inosine, thereby restoring T cell metabolic fitness and effector functions [21,22,23,24]. ADA1 activity has been implicated in alleviating adenosine-mediated suppression and supporting tumor-specific immune responses [12,14,25,26,27]. Furthermore, CD26 (dipeptidyl peptidase 4) is recognized for its role in T cell migration, invasion, and survival; CD26^high^ T cell subsets exhibit enhanced tumor infiltration, persistence, and memory formation [28]. The co-expression of ADA1 and CD26, therefore, offers a compelling avenue to simultaneously improve CAR T cell expansion, infiltration, and metabolic resistance within the hostile tumor microenvironment [14,15].

Here, we present a new generation of multi-receptor CAR T cells, termed MRCAR, that combine the tumor targeting specificity of GPC3- and HER2-directed CARs with IL-15-mediated proliferative support and metabolic rewiring through ADA1 and CD26 co-expression. We systematically evaluate the functional impact of the ADA1–CD26 axis on CAR T cell proliferation, cytotoxicity, exhaustion, invasion, and persistence in preclinical models of hepatocellular carcinoma (HCC) and breast cancer. Mechanistically, we show that ADA1 suppresses systemic toxicity by attenuating pro-inflammatory cytokine production and promoting beneficial macrophage polarization. CD26 enhances the invasive capacity of CAR T cells and facilitates tumor infiltration. Together, ADA1–CD26 rewiring confers remarkable improvements in anti-tumor efficacy and safety, providing a blueprint for the clinical development of CAR T cell therapies targeting solid tumors.

## 2. Materials and Methods

### 2.1. Cell Lines and Cell Culture

The HEK293T and HepG2 cell lines were obtained from the American Type Culture Collection (ATCC). BT-474, SUM159, MDA-MB-231, and MDA-MB-468 tumor cell lines were kindly provided by Dr. Jin Wang (Baylor College of Medicine, Houston, TX, USA). T47D and MCF7 cells were kindly provided by Dr. Xia Gao (Baylor College of Medicine, Houston, TX, USA). All cell lines were cultured in Dulbecco’s modified Eagle medium (DMEM; Corning, Corning, NY, USA) supplemented with 10% fetal bovine serum (FBS; Gibco, Wlatham, MA, USA) and maintained under standard cell culture conditions.

### 2.2. CAR Construct Design and Retroviral Production

The GPC3-CAR retroviral vector encodes an anti-human GPC3 single-chain variable fragment (scFv) derived from the GC33 clone, followed by a CD28 transmembrane domain, 4-1BB intracellular signaling domain, and CD3ζ signaling domain [19,20,29]. The HER2-specific CAR used in this study is a second-generation construct previously evaluated in clinical studies [30,31], and encodes an anti-human HER2 scFv, followed by a CD28 transmembrane domain, a CD28 intracellular signaling domain, and a CD3ζ signaling domain. To generate the ADA1.CD3scFv/CD26 vector (MR vector), codon-optimized sequences encoding CD26, T2A, and ADA1.CD3scFv were synthesized by GenScript and subcloned into an SFG γ-retroviral vector [19,29]. Retroviral particles were produced in HEK293T cells by co-transfection with packaging plasmids (RD114, PegPam3); supernatants were harvested 48 h post-transfection.

### 2.3. Primary Human T Cell Isolation and Transduction

Human peripheral blood mononuclear cells (PBMCs) purchased from STEMCELL Technologies (Vancouver, BC, Canada) were stimulated for 48 h on plates coated with OKT3 (130-093-387, Miltenyi Biotec, Auburn, CA, USA) and anti-CD28 (BD Pharmingen, San Jose, CA, USA) monoclonal antibodies in RPMI-1640 medium (Corning) supplemented with 10% FBS (Gibco) and 100 U/mL IL-2 (PeproTech, Cranbury, NJ, USA). After 48 h of stimulation, the cells were transduced on Retronectin-coated (Takara, Kusatsu, Shiga, Japan) plates preloaded with retroviral particles. Following 48 h of transduction, the cells were removed from the plates, washed, and cultured in complete RPMI-1640 medium supplemented with IL-2 for further expansion.

### 2.4. Flow Cytometry

An Alexa Fluor 647-conjugated anti-F(ab′)_2_ antibody (115-605-006, Jackson ImmunoResearch, West Grove, PA, USA) was used to detect GPC3-CAR expression. Alexa Fluor 647-conjugated HER2 protein (AFR1129, Bio-Techne, Minneapolis, MN, USA) was used to detect HER2 expression. The antibodies used for T-cell phenotyping and exhaustion analyses included CD3-BV785 (317329, BioLegend, San Diego, CA, USA), CD45RA-BV570 (304131, BioLegend), CCR7-APC (353213, BioLegend), CD62L-BV711 (304859, BioLegend), CD26-FITC (555436, BD Biosciences, San Jose, CA, USA), PD-1-APC (329908, BioLegend), LAG-3-Pacific Blue (369341, BioLegend), TIM-3-PE (565570, BD Biosciences), and TIGIT-AF700 (FAB7898N, R&D Systems, Minneapolis, MN, USA). Additional antibodies included CD80-BV605 (305227, BioLegend), HLA-DR-FITC (361706, BioLegend), CD163-BV711 (333630, BioLegend), CD206-Alexa Fluor 647 (321116, BioLegend), and HER2-Alexa Fluor 647 (324412, BioLegend). Flow cytometry data were acquired using a Cytek Aurora Spectral Analyzer (Cytek Biosciences, Fremont, CA, USA) and analyzed using FlowJo software v11 (Tree Star, Ashland, OR, USA).

### 2.5. IL-15 ELISA and ADA1 Activity Assays

To measure IL-15 concentration, the Human IL-15 ELISA Kit (R&D Systems, Cat. No. D1500, a Bio-Techne Brand, Minneapolis, MN, USA) was used according to the manufacturer’s instructions. Cell culture supernatants were collected, centrifuged, and stored frozen until analysis. For determination of ADA activity, cells were cultured at a density of 2 × 10^6^ cells/mL in 12-well plates for 24 h. Cell culture supernatants and cells were then collected for ADA activity analysis. ADA activity was measured using the Adenosine Deaminase Activity Assay Kit (Elabscience, Cat. No. E-BC-K197-M, Houston, TX, USA) according to the manufacturer’s instructions.

### 2.6. In Vitro Cytotoxicity Assays

The cytotoxicity of CAR T cells was evaluated using an LDH release assay. GPC3-CAR T cells were co-cultured with Huh7 cells, whereas HER2-CAR T cells were co-cultured with BT-474, T47D, MCF7, SUM159, MDA-MB-231, or MDA-MB-468 tumor cells at the indicated effector-to-target (E:T) ratios. After 4 or 18 h of co-culture, cell culture supernatants were collected, and LDH release was measured using the LDH Cytotoxicity WST Assay (ENZO, Cat. No. ENZ-KIT157, Farmingdale, NY, USA) according to the manufacturer’s instructions.

### 2.7. Repeated Tumor Stimulation and Exhaustion Marker Assessment

CAR T cells were repeatedly co-cultured with fresh Huh7 or HepG2 tumor cells at a 1:1 effector-to-target (E:T) ratio. After 3 days of co-culture, non-adherent CAR T cells were collected, counted, and re-stimulated with fresh target cells at the same 1:1 E:T ratio. This process was repeated for a total of three rounds of tumor stimulation. At the end of each co-culture interval, CAR T cells were harvested for assessment of cell viability and phenotype. Annexin V staining was performed using the ApoScreen Annexin V Apoptosis Kit (SouthernBiotech, Cat. No. 10010-09, Birmingham, AL, USA) according to the manufacturer’s instructions. The expression of exhaustion-associated markers, including LAG-3, PD-1, TIGIT, and TIM-3, was assessed by flow cytometry after each round of tumor stimulation.

### 2.8. CFSE Proliferation Assay

CAR T-cell proliferation was assessed using the Carboxyfluorescein Diacetate Succinimidyl Ester (CFSE) Cell Division Tracking Kit (BioLegend, Cat. No. 423801, San Diego, CA, USA). CAR T cells were labeled with CFSE according to the manufacturer’s instructions and co-cultured with Huh7 cells at a 1:1 ratio for 3 days. Following co-culture, CAR T cells were collected and analyzed by flow cytometry. T-cell proliferation was quantified based on the progressive dilution of CFSE fluorescence resulting from cell division.

### 2.9. Mitochondrial Membrane Potential Analysis

Mitochondrial membrane potential was assessed using the MitoProbe™ TMRM Kit for Flow Cytometry (Thermo Fisher Scientific, Cat. No. M20036, Waltham, MA, USA) according to the manufacturer’s instructions. Briefly, cells were resuspended in PBS and stained with TMRM at a final concentration of 20 nM and incubated for 30 min at 37 °C in 5% CO_2_, protected from light. Following staining, cells were optionally washed once and resuspended in PBS. TMRM fluorescence was measured by flow cytometry using 561-nm excitation and a PE-equivalent emission filter.

### 2.10. HER2-ADC Resistance Assay

To evaluate the sensitivity of tumor cells to HER2 antibody–drug conjugate (HER2-ADC), each tumor cell line was treated with HER2-ADC (MedChemExpress, Cat. No. HY-138298, Monmouth Junction, NJ, USA) at concentrations of 0, 0.1, 1, or 10 µg/mL. After 3 or 5 days of incubation, cell viability was assessed using the Cell Counting Kit-8 (CCK-8) assay (Boster, Cat. No. AR1160, Pleasanton, CA, USA) according to the manufacturer’s instructions.

### 2.11. Transwell Invasion Assay

24-well Transwell inserts with 3.0-µm or 5.0-µm pores (Cat. No. 353096, Cat. No. 3421, Corning Incorporated, Corning, NY, USA) were coated with Matrigel (Cat. No. 354234, Corning Incorporated, Corning, NY, USA) and allowed to dry before reconstitution with serum-free medium. A total of 2 × 10^5^ cells were added to each Transwell insert. The inserts were placed in wells containing 0.4 mL of serum-free medium supplemented with 20 nM SDF-1α. For inhibitor studies, cells were pre-incubated for 1 h with a CD45 inhibitor (Sigma-Aldrich, Cat# 540215, St. Louis, MO, USA), p38 MAPK inhibitor SB203580 (Sigma-Aldrich, Cat# 559389), MEK1 inhibitor PD98059 (Sigma-Aldrich, Cat# 513000), or PI3K inhibitor LY294002 (Promega Corporation, Cat# V1201, Madison, WI, USA). After 24 h of incubation, cells that had migrated through the Matrigel-coated membrane were collected and counted using a hemocytometer. The total cell number was determined from cells cultured under the same inhibitor treatment conditions without Transwell inserts. The invasion level was calculated as the number of cells that migrated through the Matrigel-coated membrane divided by the total cell number [32,33,34].

### 2.12. RT-qPCR

Total RNA was extracted using RNeasy Plus Mini Kit (50) (Qiagen, Cat# 74134, Germantown, MD, USA) and reverse-transcribed using qScript cDNA SuperMix (Quantabio, Cat# 95048-100, Beverly, MA, USA). Quantitative PCR was carried out with SsoAdvanced Universal SYBR Green Supermix (Bio-Rad, Hercules, CA, USA, Cat# 1725270) on a QuantStudio™ 6 Flex Real-Time PCR System (Thermo Fisher Scientific). The following primers were used for detection: mouse GAPDH-F (CATGGCCTTCCGTGTTCCTA) and GAPDH-R (CCTGCTTCACCACCTTCTTGAT); human HER2scfv-F (AACACGGCCTACCTGCAAAT) and HER2scfv-R (ACAGGAACTTGTGGCTCTGG).

### 2.13. Western Blot Analysis of SDF-1α-Induced Signaling

To assess SDF-1α-induced phosphorylation, cells were cultured overnight in serum-free medium and stimulated with SDF-1α at a final concentration of 10 nM for 2.5- or 10-min [35,36,37]. Cells were then diluted in ice-cold PBS containing 1 mM sodium vanadate and harvested. Cells were lysed in RIPA buffer supplemented with Mini Protease Inhibitor Cocktail (Roche, Indianapolis, IN, USA) and Phosphatase Inhibitor Cocktail Set (Millipore, Burlington, MA, USA). Protein concentrations in the cell lysates were determined using the BCA Protein Assay Kit (Life Technologies, Cat. No. 23227, Waltham, MA, USA). Equal amounts of protein were separated by 10% SDS-PAGE and transferred to PVDF membranes (Bio-Rad) using standard procedures. Membranes were probed with primary antibodies against Akt (Cell Signaling Technology, Cat. No. 9272S, Danvers, MA, USA), phospho-Akt (Ser473) (Cell Signaling Technology, Cat. No. 9271T), p44/42 MAPK (ERK1/2) (Cell Signaling Technology, Cat. No. 9102S), and phospho-p44/42 MAPK (ERK1/2) (Cell Signaling Technology, Cat. No. 9101S).

### 2.14. In Vivo Tumor Models

NSG mice (NOD.Cg-Prkdc^scid Il2rg^tm1Wjl/SzJ, Jackson Labs Cat# 005557, Bar Harbor, ME, USA), female and male, 6–8 weeks. All animal studies were approved by the Institutional Animal Care and Use Committee (IACUC 2023-0279 HD and IACUC 2024-0221 HD). Huh7, Huh7-Luc, or T47D tumor cells were resuspended in 100 μL of PBS and administered to mice by subcutaneous (s.c., flank) or intraperitoneal (i.p.) injection, as indicated. Tumors were allowed to establish and grow to approximately 100 mm^3^, after which mice were randomized into treatment groups. Mice bearing tumors in the indicated models received a single intravenous (i.v.) or intraperitoneal (i.p.) injection of the indicated number of CAR T cells. Tumor growth was monitored by caliper measurement three times per week, and tumor volume was calculated using the formula: tumor volume = (length × width^2^)/2. Mice were monitored daily for tumor progression and overall health throughout the study. Humane endpoints included tumor size >1.5 cm^3^ or signs of distress.

### 2.15. In Vivo Imaging

Huh7 or CAR T cells were transduced with a retroviral vector encoding enhanced firefly luciferase (effLuc), which we generated in-house as previously described [19,20,29]. Mice received 150 mg/kg D-luciferin (GoldBio Cat# LUCNA, St. Louis, MO, USA) intraperitoneally, and bioluminescent signal was acquired using PerkinElmer Living Image software (Version 4.7.4, Waltham, MA, USA). Signal quantification used photon flux (p/s/cm^2^/sr); background was deducted from each ROI.

### 2.16. Cytokine Multiplex Analysis

Mouse serum was collected from blood on days 1, 2, and 7 after CAR T cell administration, and stored at −80 °C. Cytokine concentrations in the serum were measured in duplicate using Luminex bead-based assays (LXSAHM-09 kits, Bio-Techne, Minneapolis, MN, USA) following the manufacturer’s instructions specific to each kit. Briefly, serum samples were thawed on ice, centrifuged, and diluted 1:2 with RD6-52. Diluted samples were incubated with beads for 2 h at room temperature with shaking, followed by incubation with Biotin-Antibody Cocktail and streptavidin-PE. Samples were analyzed using the Bio-Plex 200 system and Bio-Plex Manager 7.2 software (Bio-Rad, Hercules, CA, USA). Fifty beads per analyte were collected with a 60-s timeout set for plate analysis. Cytokine concentrations were calculated using a standard curve with a 5-parameter logistic-fitting method in Bio-Plex manager software (Version 6.2, Hercules, CA, USA), and the average values of the replicates were used for the final analysis [38,39,40].

### 2.17. Macrophage Co-Culture and Polarization Assays

Human monocytes were isolated from PBMCs and differentiated into macrophages with M-CSF (50 ng/mL, PeproTech Cat# 300-25) for 7 days. To assess macrophage polarization, macrophages were polarized toward an M1-like phenotype by treatment with LPS (100 ng/mL) and IFN-γ (20 ng/mL), whereas M2 polarization was induced with IL-4 (20 ng/mL) and IL-13 (20 ng/mL). Macrophage polarization was evaluated by assessing the expression of M1-associated markers (CD80, CD86, and HLA-DR) and M2-associated markers (CD163 and CD206) by flow cytometry. Following polarization, macrophages were co-cultured with CAR T cells at an E:T ratio of 1:1 for 48 h.

### 2.18. Statistical Analysis

Results are expressed as mean ± SEM or SD as indicated. Sample sizes (*n*) and number of replicates are described in figure legends. Two-tailed unpaired Student’s *t*-test for two-group comparisons. One-way or two-way ANOVA with Tukey’s or Bonferroni post-hoc corrections for multi-group comparisons. Log-rank (Mantel–Cox) test for Kaplan–Meier survival curves. *p*-values < 0.05 were considered significant. GraphPad Prism v9.0 was used for all analyses.

## 3. Results

### 3.1. Engineering and Functional Characterization of Multi-Receptor CAR T Cells

**Figure 1:**
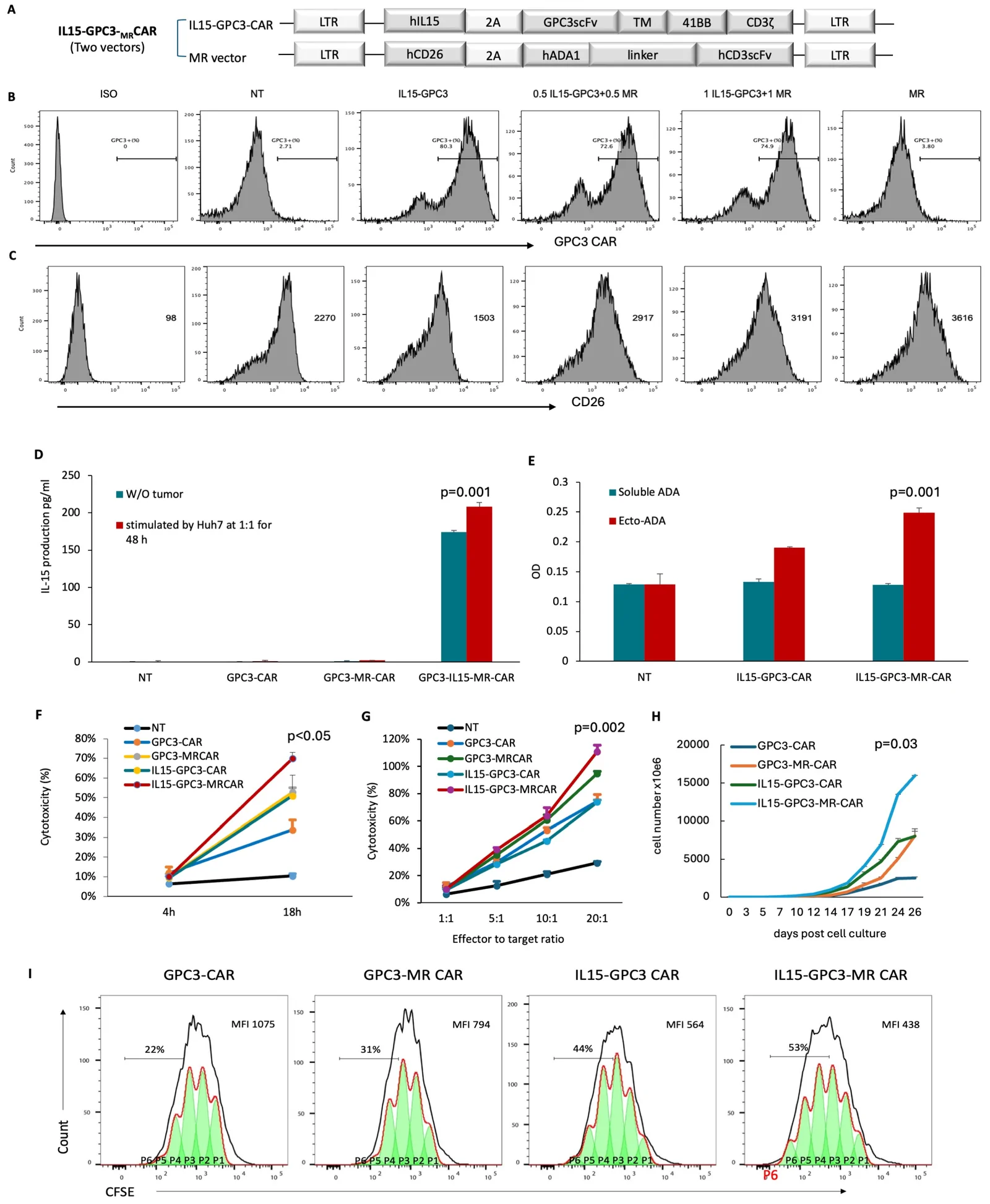
Molecular and functional characterization of IL15-GPC3-MRCAR T cells with ADA1 and CD26 co-expression. (**A**) Schematic illustration of the IL15-GPC3-MR CAR T-cell engineering strategy. CAR T cells were generated using a two-vector retroviral transduction system consisting of a CAR vector and a separate MR retroviral vector encoding CD26 and ADA1-CD3scFv. (**B**) Representative flow cytometric analysis of GPC3-CAR expression in NT (non-transduced), GPC3, GPC3-MR, IL15-GPC3, and IL15-GPC3-MR CAR T-cell products following retroviral transduction (data are representative of *n* = 5 independent biological experiments). (**C**) Representative flow cytometric analysis of CD26 expression in the indicated CAR T-cell products (data are representative of *n* = 5 independent biological experiments). (**D**) CAR T cells were co-cultured with or without Huh7 cells at an effector-to-target ratio of 1:1 for 48 h. IL-15 levels in the culture supernatants were quantified by ELISA (data are presented as mean ± SEM from *n* = 3 independent biological experiments; statistical significance was determined using a two-way ANOVA with Tukey’s multiple comparisons test comparing CAR T cells with versus without Huh7 co-culture for each group). (**E**) IL15-GPC3 CAR T cells transduced with or without the MR vector (2 × 10^6^ cells/ml) were cultured for 24 h. Culture supernatants and cells were then analyzed for ADA enzyme activity according to the manufacturer’s instructions (data are presented as mean ± SEM from *n* = 3 independent biological experiments; statistical significance was evaluated using a two-tailed unpaired Student’s *t*-test comparing IL15-GPC3 CAR to IL15-GPC3-MRCAR). (**F**) The cytotoxic activity of CAR T cells against Huh7 cells at a 1:1 E:T ratio was evaluated using an LDH release assay at 4 and 18 h (data are presented as mean ± SEM from *n* = 3 independent biological experiments; statistical comparisons between groups were performed using a two-way ANOVA with Bonferroni’s multiple comparisons test comparing all five groups at the 18 h time point). (**G**) Cytotoxicity was further assessed after 4 h of coculture at increasing E:T ratios of 1:1, 5:1, 10:1, and 20:1 (data are presented as mean ± SEM from *n* = 3 independent biological experiments; analyzed via two-way ANOVA with Tukey’s multiple comparisons test comparing all constructs at each ratio). (**H**) CAR T cells were expanded in vitro, and cell numbers were determined at indicated time points using a hemocytometer. Cell expansion was presented as a growth curve (data are presented as mean ± SEM from *n* = 3 independent biological experiments; analyzed using a two-way repeated-measures ANOVA comparing groups across time points). (**I**) CAR T cells were labeled with CFSE and co-cultured with Huh7 cells at a 1:1 E:T ratio for 3 days. CAR T-cell proliferation was then assessed by flow cytometry based on CFSE dilution. Data shown are from a single representative experiment (*n* = 1), with consistent findings previously established using related CAR-T systems [14].

The immunosuppressive microenvironment of solid tumors presents multiple barriers to effective CAR T cell therapy, including poor persistence, limited expansion, and functional exhaustion. To address these challenges, we engineered multi-receptor CAR T cells (MRCAR) co-expressing adenosine deaminase 1 (ADA1), dipeptidyl peptidase 4 (CD26), and IL-15, with glypican-3 (GPC3) CARs (Figure 1A). In this study, ADA1 is membrane-anchored via an anti-CD3 scFv to restrict its activity to the T cell surface, as our previous work showed that secreted ADA1 can induce tumor cell proliferation in vitro [14]. Retroviral transduction of primary human T cells from healthy donors resulted in effective expression of both GPC3-CAR and CD26, confirmed by flow cytometry (Figure 1B,C). While primary human T cells display baseline endogenous expression of CD26, precluding a discrete double-positive flow cytometry gate, successful co-transduction of the two-vector system was verified by high CAR expression (>70%) alongside a clear MFI shift for CD26. IL-15 was readily detected in supernatants of GPC3-IL15-MR CAR T cell cultures (Figure 1D). ADA enzymatic activity assays demonstrated that MR CAR T cells secreted higher levels of ADA, which in turn bound to the surface of CAR T cells, as observed in our previous studies [19,29]. Compared to control conditions, there was a clear increase in ADA activity associated with MR CAR T cells, indicating enhanced ADA binding to the cell surface (Figure 1E).

In functional assays, MR CAR T cells demonstrated greater cytotoxicity than conventional CAR T cells at an E:T ratio of 1:1 following 18 h of coculture with Huh7 cells (Figure 1F). Comparable cytotoxicity was observed across the constructs at lower effector-to-target ratios, with modest enhancements becoming apparent at higher ratios (Figure 1G), indicating that metabolic and migratory rewiring supports preserved anti-tumor function. While IL15 expression enhanced the expansion of GPC3 CAR T cells, incorporation of the MR module further promoted CAR T-cell expansion, resulting in greater expansion compared with conventional CAR T cells (Figure 1H). Consistent with our previous observations in analogous CAR-T models [14], a representative CFSE dilution assay demonstrated robust CAR T-cell proliferation after 3 days of co-culture with Huh7 cells (Figure 1I).

### 3.2. ADA1–CD26 MRCAR T Cells Resist Functional Exhaustion Following Repeated Tumor Antigen Stimulation

**Figure 2:**
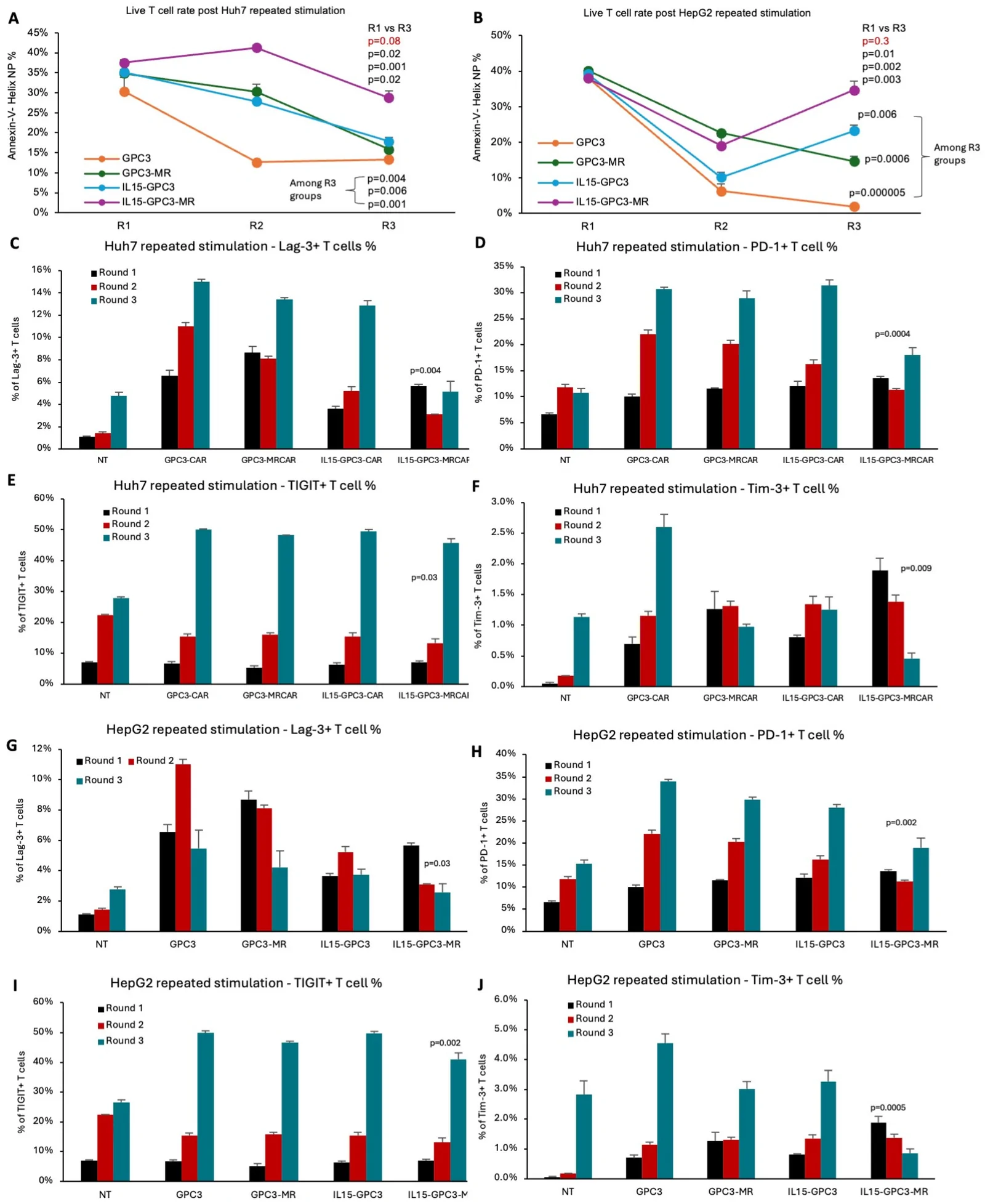
IL15-GPC3-MRCAR T cells demonstrate reduced exhaustion following repeated tumor antigen exposure in vitro. (**A**,**B**) Quantification of live CAR T cell recovery after three rounds of antigen stimulation with Huh7 (**A**) or HepG2 (**B**) cells. Data shown as percentage of live CD3+ T cells (presented as mean ± SEM from *n* = 3 independent biological experiments; statistical significance was determined using a two-way ANOVA followed by Tukey’s multiple comparisons test, comparing all four groups). (**C**–**F**) Expression of exhaustion markers LAG-3 (**C**), PD-1 (**D**), TIGIT (**E**), and TIM-3 (**F**) in CAR T cells after Huh7 stimulation, measured by flow cytometry. (**G**–**J**) Same exhaustion marker analysis after repeated exposure to HepG2 cells (mean ± SEM, *n* = 3). MRCAR T cells exhibited significantly lower exhaustion marker expression versus conventional CAR T cells. For panels (**C**–**J**), data are presented as mean ± SEM from *n* = 3 independent biological experiments; statistical significance was determined using a two-way ANOVA with Tukey’s multiple comparisons test, comparing all five groups across each round of stimulation.

To determine whether co-expression of ADA1 and CD26 could preserve CAR T cell fitness under conditions of sustained antigen stimulation, MRCAR T cells and conventional CAR T cells were subjected to three consecutive rounds of co-culture with Huh7 or HepG2 hepatocellular carcinoma cells. Following each round of tumor-cell exposure, viable T cells were recovered and evaluated to assess their capacity to withstand repeated antigenic challenge. MRCAR T cells demonstrated substantially greater persistence throughout serial stimulation (Figure 2A,B). The progressive preservation of viable T cells in the MRCAR T cell group suggests that ADA1–CD26 expression may protect against the cumulative loss of cellular fitness associated with chronic antigen exposure. In contrast, conventional CAR T cells exhibited a pronounced decline in viability with successive rounds of stimulation, consistent with the development of an exhausted or dysfunctional phenotype.

**Figure 3:**
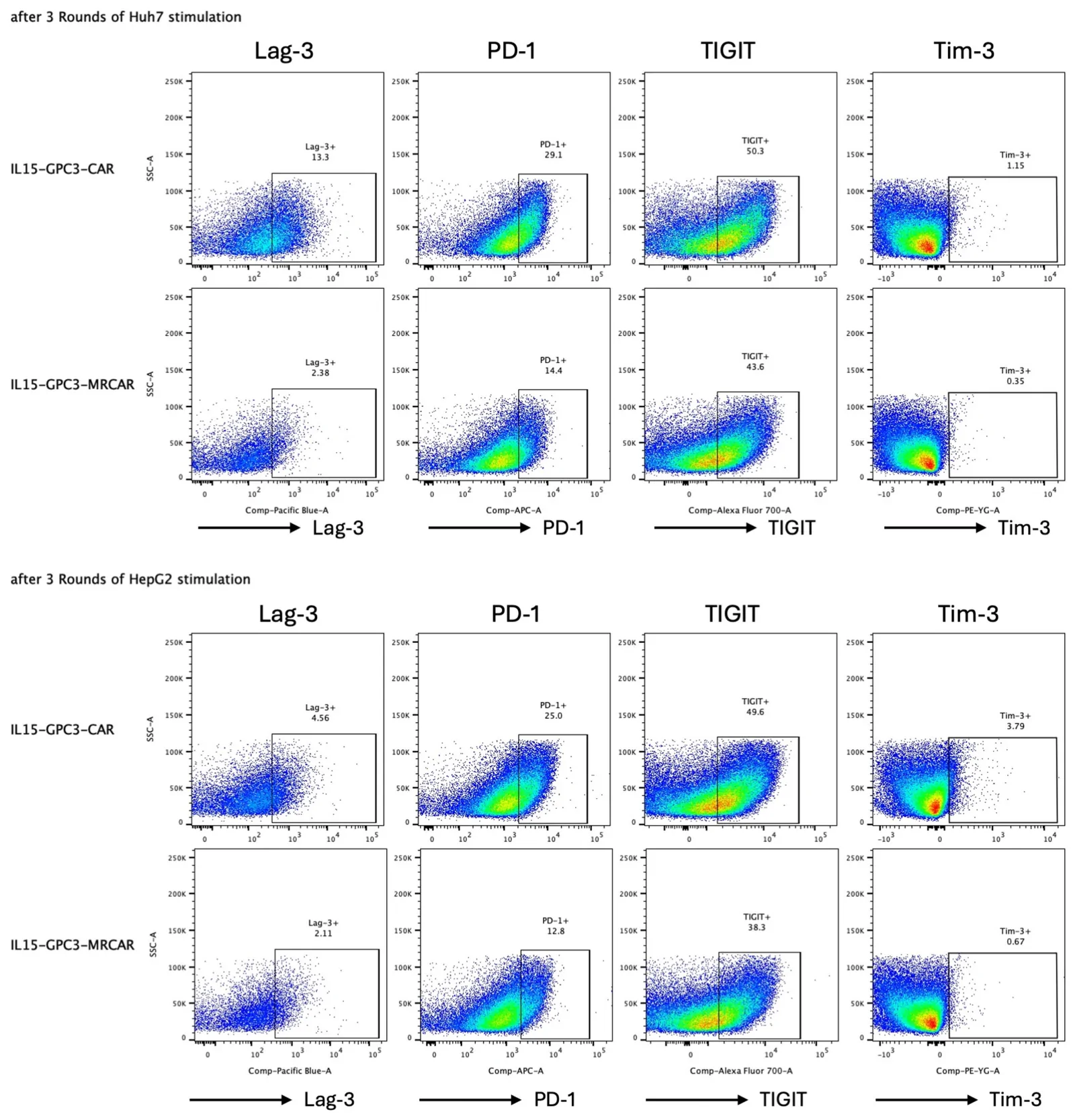
Representative flow cytometry plots of exhaustion marker LAG-3, PD-1, TIGIT, TIM-3 expression following repeated co-culture. IL15-GPC3-CAR and IL15-GPC3-MRCAR T cells were evaluated after three rounds of co-culture with Huh7 and HepG2 tumor cell lines. Representative flow cytometry gating and staining data show the expression levels of key exhaustion markers on the respective T cell populations, complementing the summarized quantitative data presented in Figure 2.

To characterize MRCAR T cells further, the expression of established inhibitory and exhaustion-associated receptors was examined by flow cytometry following repeated tumor-antigen stimulation. The results suggest a potential reduction in exhaustion markers LAG3, PD-1, TIGIT, and TIM3 compared with conventional CAR T cells (Figure 2C–F). Importantly, the protective phenotype was not restricted to a single tumor model. Similar reductions in exhaustion-marker expression and improved T cell persistence were observed when MRCAR T cells were repeatedly challenged with HepG2 cells, demonstrating that the effect was reproducible across two hepatocellular carcinoma cell lines (Figure 2G–J). Summary data illustrating exhaustion marker expression on IL15-GPC3-CAR versus IL15-GPC3-MRCAR T cells following three rounds of co-culture with Huh7 and HepG2 targets are presented in Figure 2C–J, with representative flow cytometry staining data provided in Figure 3. Thus, the enhanced resistance of MRCAR T cells to serial antigen stimulation appears to reflect an intrinsic property of the ADA1–CD26-modified CAR T cell population rather than a tumor-cell-line-specific phenomenon.

### 3.3. CD26 Expression Enhances CAR T Cell Tumor Invasion Through MAPK and PI3K Pathways

**Figure 4:**
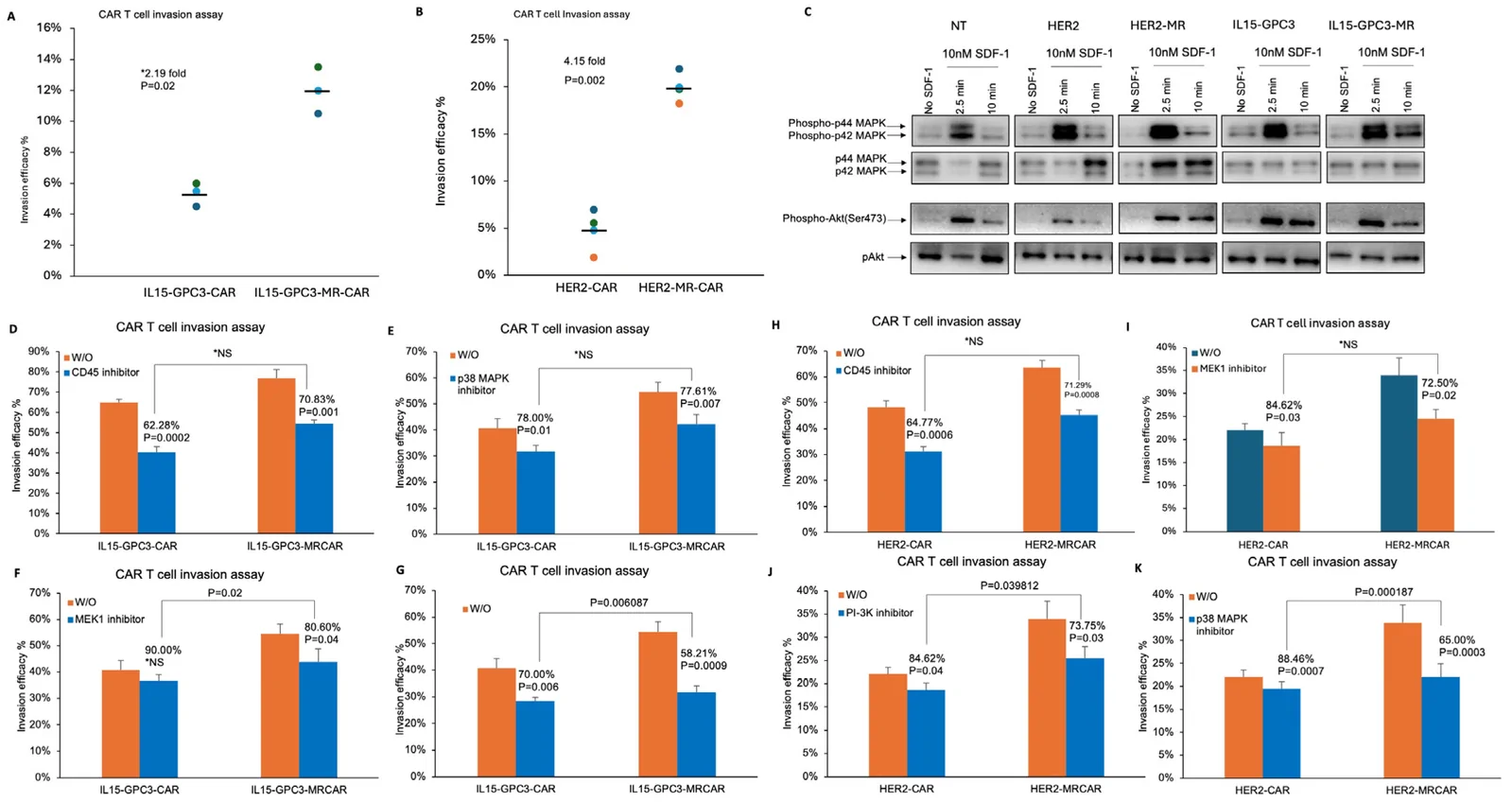
CD26 expression enhances CAR T cell invasion via MAPK and PI3K signaling. (**A**,**B**) Transwell invasion assays were performed using IL15-GPC3-CAR T cells (**A**) and HER2-CAR T cells (**B**). The percentage of invasion was calculated as the number of cells that migrated through the Transwell membrane divided by the total cell number (mean ± SEM, *n* = 3, * indicates the fold of change). (**C**) Western blotting showed that SDF-1α induced phosphorylation of signaling proteins in CAR T cells. CAR T cells were incubated with SDF-1α, and the activation of signaling kinases was assessed by measuring their phosphorylation levels. Data shown are from a single preliminary experiment (*n* = 1). (**D**–**G**) IL15-GPC3 CAR T-cell invasion was assessed following pretreatment with kinase inhibitors. Cells were incubated with the indicated inhibitors for 1 h before the initiation of the invasion assay. The CD45 inhibitor (**D**) was used at 6 μM, the p38 MAPK inhibitor SB203580 (**E**) at 10 μM, and the MEK1 inhibitor PD98059 (**F**) and PI3K inhibitor LY294002 (**G**) at 50 μM. (**H**–**K**) HER2-CAR T-cell invasion was assessed following the same inhibitor treatments. For panels (**A**,**B**,**D**–**K**), individual data points and/or data are presented as mean ± SEM from *n* = 3 independent biological experiments (* indicates NS, no significance). Statistical significance was determined using a two-tailed unpaired Student’s *t*-test comparing the control group versus the treated/CD26-expressing group.

Since CD26 has been implicated in T cell migration, we assessed its impact on CAR T cell invasiveness in vitro and in vivo. In Matrigel-coated transwell assays, GPC3-targeted MRCAR T cells, which exhibited higher levels of CD26 expression, demonstrated significantly greater invasive activity in response to SDF-1α compared with conventional CAR T cells (Figure 4A). Parallel migration enhancement was noted for HER2-targeted MRCAR T cells (Figure 4B).

Phosphorylation of several signaling proteins has been shown to play important roles in cell migration and invasion in various cell types [35,41,42]. To investigate the signaling pathways underlying CD26-regulated CAR T-cell invasion, we evaluated the phosphorylation status of Akt and p44/42 MAPK (ERK1/2) following SDF-1α stimulation. CAR T cells were stimulated with SDF-1α for 2.5 or 10 min, and the phosphorylation of Akt and p44/42 MAPK was assessed by Western blotting. As a preliminary observation, a single experiment (*n* = 1) showed that SDF-1α stimulation induced phosphorylation of both Akt and p44/42 MAPK in CAR T cells, with increased phosphorylation observed following stimulation compared with unstimulated cells (Figure 4C). Although housekeeping protein controls were not included in this analysis, protein quantification was performed to ensure equal loading across samples. Visual inspection of the Western blot figures shows that baseline signal intensity (prior to SDF-1α stimulation) is similar across groups, indicating consistent protein loading and transfer. These findings demonstrate that SDF-1α activates the Akt and p44/42 MAPK signaling pathways in CAR T cells, suggesting that these pathways may contribute to SDF-1α-mediated CAR T-cell invasive activity.

To investigate the signaling pathways underlying CD26-mediated enhancement of CAR T cell migration, we pre-incubated CAR T cells with selective inhibitors targeting PI3K, MEK1, p38 MAPK, or CD45 and evaluated their invasive capacity in vitro (Figure 4D–K). Inhibition of PI3K with LY294002 significantly reduced the invasive activity of both GPC3- and HER2-targeted MRCAR T cells, with a significantly greater reduction relative to their corresponding conventional CAR T cells, suggesting a conserved role for PI3K signaling in CD26-enhanced invasion. In the GPC3-targeted model, MEK1 inhibition with PD98059 similarly produced a significant differential reduction in invasion between IL15-GPC3-CAR and IL15-GPC3-MRCAR T cells, whereas inhibition of p38 MAPK or CD45 did not significantly distinguish the two cell populations. Conversely, in HER2-targeted CAR T cells, p38 MAPK inhibition significantly reduced the enhanced invasive activity of MRCAR T cells relative to conventional CAR T cells, while MEK1 and CD45 inhibition did not produce a significant differential effect. These findings suggest that PI3K signaling represents a common pathway associated with CD26-mediated CAR T-cell invasion, whereas MEK1 and p38 MAPK may contribute in a tumor antigen/CAR-dependent manner.

### 3.4. ADA1–CD26 Axis Promotes CAR T Cell Persistence and Tumor Control in Hepatocellular Carcinoma

**Figure 5:**
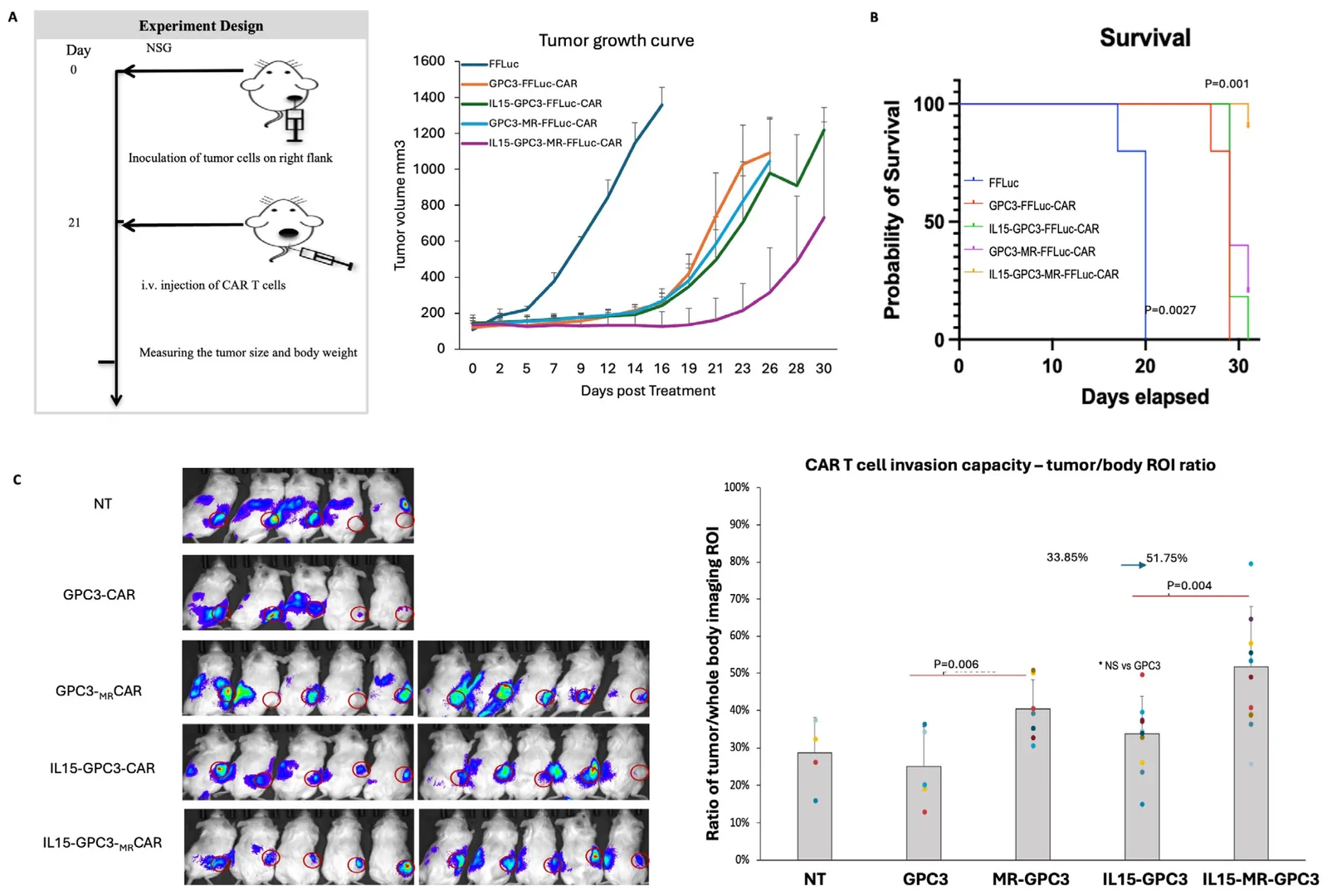
ADA1–CD26 axis improves therapeutic efficacy and persistence in Huh7 hepatocellular carcinoma mouse models. A murine HCC xenograft model was established in NSG mice by subcutaneous inoculation of 2 × 10^6^ Huh7 tumor cells into the right flank. When the average tumor volume reached approximately 100 mm^3^, mice were treated with 1 × 10^7^ FFLuc, GPC3-FFLuc-CAR, GPC3-MR-FFLuc-CAR, IL15-GPC3-FFLuc-CAR, or IL15-GPC3-MR-FFLuc-CAR T cells. (**A**) Tumor volume was monitored every 2–3 days (data are presented as mean ± SEM from *n* = 5–10 mice per group; statistical differences were determined using a two-way ANOVA with Tukey’s multiple comparisons test comparing all treatment groups across time points). (**B**) Mouse survival was monitored throughout the study (survival data from *n* = 5–10 mice per group; statistical significance was determined using the log-rank Mantel–Cox test comparing the IL15-GPC3-MR-FFLuc-CAR group against the IL15-GPC3-FFLuc-CAR control group). (**C**) At one day post T cell i.v. administration, IVIS imaging was performed to assess the infiltration of T cells into tumors, and photon counts were quantified (individual data points and mean ± SEM are shown from *n* = 5–10 mice per group; statistical significance was evaluated using a one-way ANOVA followed by Tukey’s multiple comparisons test comparing all treatment groups; * no significance between IL15-GPC3 and GPC3 group).

**Figure 6:**
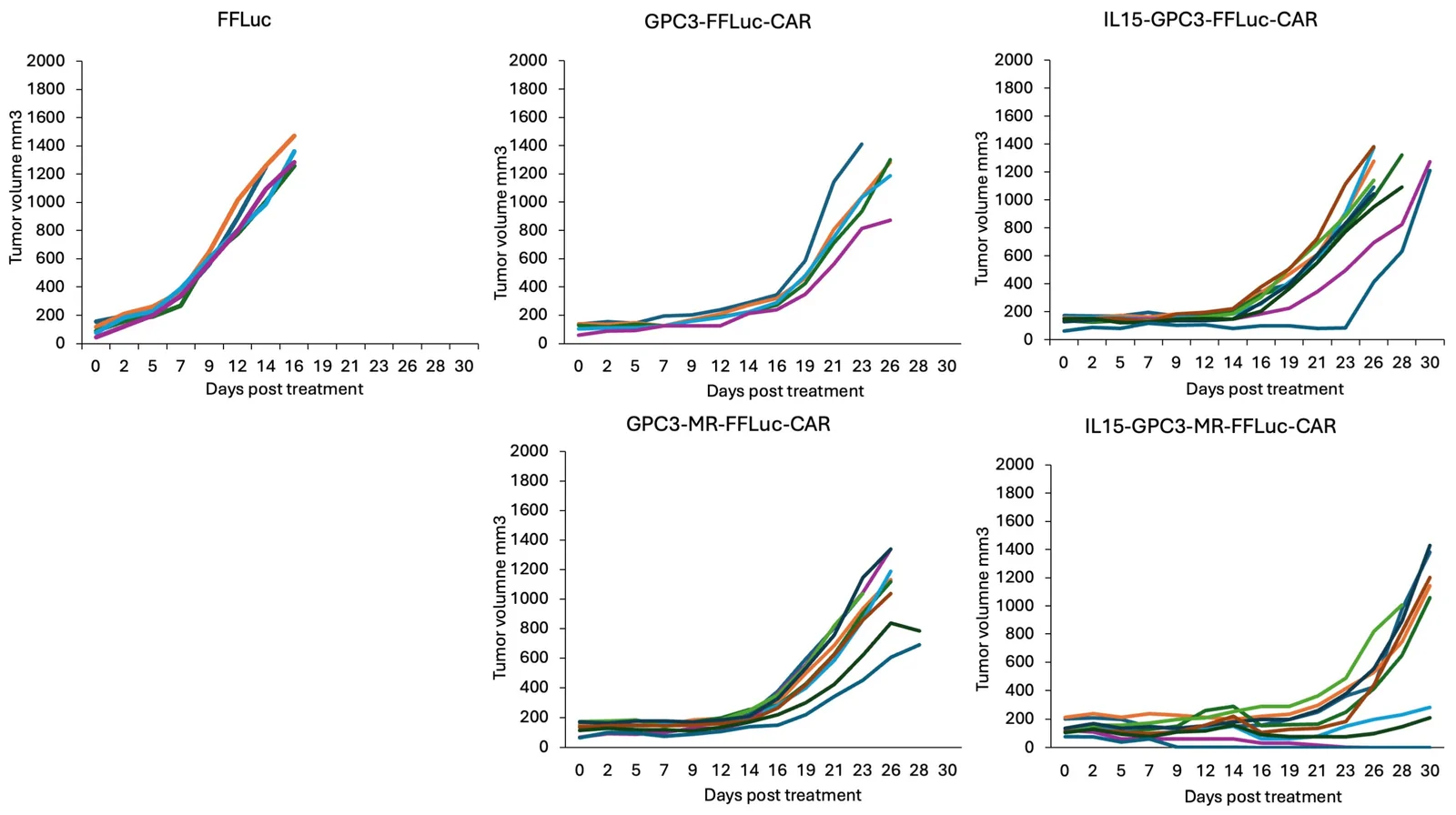
ADA1–CD26 axis improves therapeutic efficacy in Huh7 hepatocellular carcinoma mouse models. A murine HCC xenograft model was established in NSG mice by subcutaneous inoculation of 2 × 10^6^ Huh7 tumor cells into the right flank. When the average tumor volume reached approximately 100 mm^3^, mice were treated with 1 × 10^7^ FFLuc, GPC3-FFLuc-CAR, GPC3-MR-FFLuc-CAR, IL15-GPC3-FFLuc-CAR, or IL15-GPC3-MR-FFLuc-CAR T cells. Tumor volume was monitored every 2–3 days, and data are presented as individual tumor growth curves.

To determine whether ADA1–CD26 metabolic rewiring enhances these properties in vivo, MRCAR T cells were evaluated in a subcutaneous Huh7 xenograft model (*n* = 5 or 10/group). Tumor volume comparison at earlier time points (day 23/26) with sufficient group sizes confirmed lower mean/median tumor volume in the IL15-GPC3-MR-FFLuc-CAR group compared to the IL15-GPC3-FFLuc-CAR group (Mann–Whitney U test, *p* < 0.05, Figure 5A), indicating enhanced inhibition of tumor growth. At day 30, the IL15-GPC3-MR-FFLuc-CAR group showed 10 surviving mice compared to 2 mice in the IL15-GPC3-FFLuc-CAR group (log-rank *p* = 0.001, Figure 5B), though the overall separation across all four constructs remains modest, pointing to a subtle therapeutic advantage. For additional detail, individual tumor growth curves for each mouse are presented in Figure 6.

To assess whether enhanced tumor control was associated with improved CAR T cell accumulation, tumor localization was evaluated by IVIS imaging. MRCAR T cells exhibited greater accumulation at tumor sites than conventional CAR T cells and control groups. Quantitative ROI analysis showed significantly higher tumor-associated signal in the IL15-GPC3-MR CAR T-cell group than in the conventional CAR T-cell group (Figure 5C). Together, these findings indicate that ADA1–CD26 enhances CAR T cell persistence and tumor accumulation, resulting in improved antitumor efficacy and survival in HCC.

### 3.5. ADA1–CD26 Enables Sustained CAR T Cell Memory and Rechallenge Immunity in Breast Cancer Models

**Figure 7:**
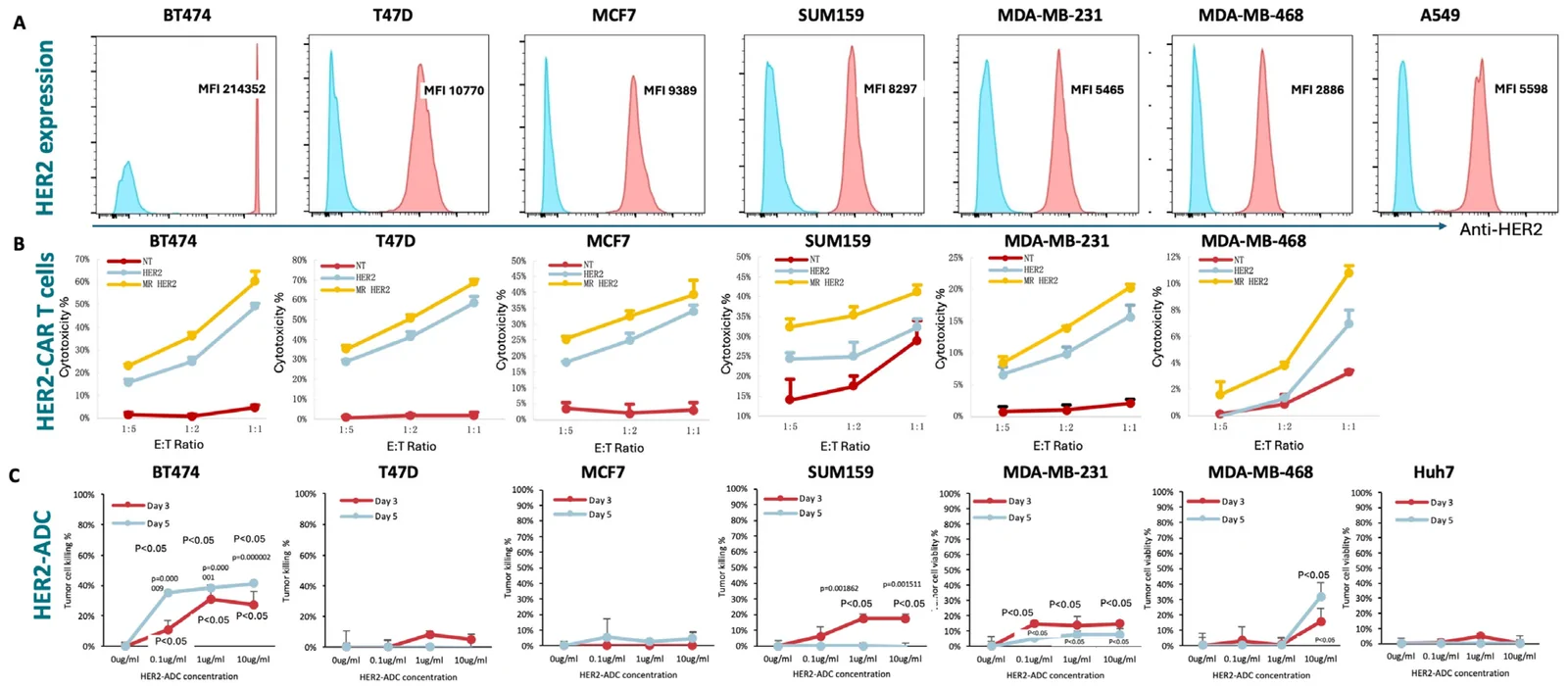
HER2-CAR T cells exhibit cytotoxicity against HER2-ADC-resistant tumor cell lines. (**A**) Flow cytometric profiling of HER2 expression in various human breast cancer lines (data are representative of *n* = 1 independent experiment). (**B**) The cytotoxic activity of CAR T cells against breast cancer cell lines at the indicated effector-to-target ratios was evaluated using an LDH release assay 24 h after coculture (data are presented as mean ± SEM from *n* = 3 independent biological experiments; statistical significance was determined using a two-way ANOVA followed by Tukey’s multiple comparisons test comparing all tested groups across the E:T ratios). (**C**) The in vitro cytotoxic effects of HER2 antibody–drug conjugate (ADC) treatment on breast cancer cell lines were evaluated using a CCK-8 assay (data are presented as mean ± SEM from *n* = 3 independent biological experiments; statistical differences were evaluated using a one-way ANOVA with Tukey’s multiple comparisons test comparing all treatment groups).

To investigate the antitumor activity of HER2-targeted MRCAR T cells, we first profiled HER2 expression across a panel of breast cancer cell lines (Figure 7A). MRCAR T cells exhibited potent cytotoxic activity against HER2-expressing tumor cells, with cytotoxicity correlating with HER2 expression levels (Figure 7B). Notably, the breast cancer cell lines displayed variable sensitivity to HER2-targeted antibody–drug conjugate (ADC) treatment (Figure 7C). This heterogeneity may reflect differences in the expression of other HER family members, such as EGFR and HER3, which can form EGFR–HER2 or HER2–HER3 heterodimers and potentially alter HER2 internalization and subsequent ADC uptake [43,44]. These findings suggest that HER2 expression alone may not fully predict sensitivity to HER2-directed ADC therapy, whereas MRCAR T cells retain potent cytotoxic activity against HER2-expressing tumor cells.

**Figure 8:**
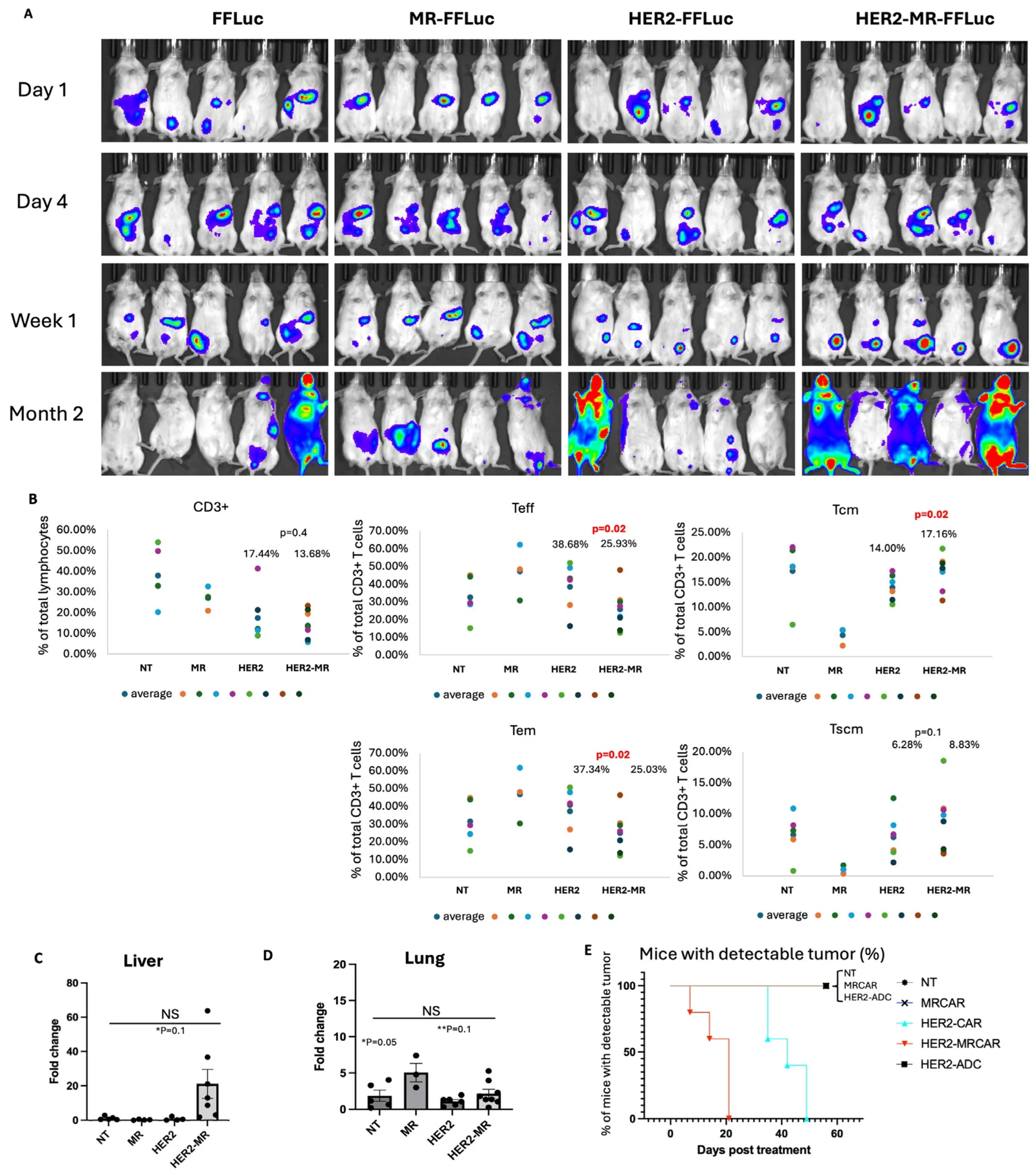
ADA1–CD26 promotes CAR T cell persistence and confers tumor rechallenge protection in breast cancer models. (**A**) Longitudinal IVIS imaging of CAR T cell persistence in T47D breast tumor xenografts. 5 × 10^6^ T47D tumor cells were injected into the fourth mammary fat pad, followed by intravenous administration of 2 × 10^6^ FFLuc, MR-FFLuc, HER2-FFLuc, or HER2-MR-FFLuc CAR T cells (data are presented as mean ± SEM from *n* = 5 mice per group). (**B**) Single-cell suspensions from mouse spleens were stained and analyzed by flow cytometry to characterize T-cell subsets, including effector (Teff, CD62L^−^CCR7^−^), central memory (Tcm, CD45RA^−^CD62L^+^CCR7^+^), effector memory (Tem, CD45RA^−^CD62L^−^CCR7^−^), and stem cell-like memory (Tscm, CD45RA^+^CD62L^+^CCR7^+^) T cells. Data are presented as mean ± SEM from *n* = 5 mice per group; statistical differences were evaluated using a two-tailed unpaired Student’s *t*-test comparing HER2-CAR versus HER2-MR-CAR groups (**C**,**D**) Tissue distribution of CAR T cells in liver and lung 3 months after CAR T-cell treatment was analyzed by qPCR (data are presented as mean ± SEM from *n* = 3–8 mice per group; statistical significance was determined using a two-tailed unpaired Student’s *t*-test comparing the indicated groups). (**E**) Tumor-bearing mice curve post-CAR T therapy (data from *n* = 5 mice per group; statistical differences between survival curves were determined using the log-rank Mantel–Cox test comparing HER2-CAR against the HER2-MR-CAR group).

CAR T cell persistence was subsequently monitored by IVIS following infusion into T47D breast cancer xenografts. HER2-MR-FFLuc CAR T cells showed greater accumulation at tumor sites at both early and late time points compared with conventional CAR T cells. Quantitative analysis demonstrated significantly higher photon flux in the MRCAR group (*n* = 5, Figure 8A). Phenotypic analysis further revealed a modest increase in the proportion of central memory (Tcm) T cells in MRCAR T cells (Figure 8B), while stem memory (Tscm) proportions remained comparable, pointing toward a slight shift in T cell phenotype. Of note, the reduction in splenic CD3+ T cells following CAR transduction (Figure 8B) likely reflects the redistribution of retroviral vector transduction-activated T cells away from secondary lymphoid organs and into systemic/peripheral tissues. CAR T-cell distribution in the liver and lung was further assessed by qPCR, with no significant differences in CAR T-cell abundance among the groups (Figure 8C,D).

Consistent with these findings, MRCAR treatment produced earlier and more sustained tumor control in the primary therapeutic model. Tumor-free status was achieved by approximately day 21 in the HER2-MRCAR group compared with day 49 in the HER2-CAR group, whereas tumors continued to grow in the HER2-ADC-treated group through day 54 (*p* < 0.01, Figure 8E). Collectively, these results indicate that ADA1–CD26 rewiring promotes CAR T cell persistence and memory-associated phenotypes, supporting durable antitumor activity in breast cancer models.

### 3.6. ADA1–CD26 Axis Attenuates Systemic Toxicity and Modulates Macrophage Polarization

**Figure 9:**
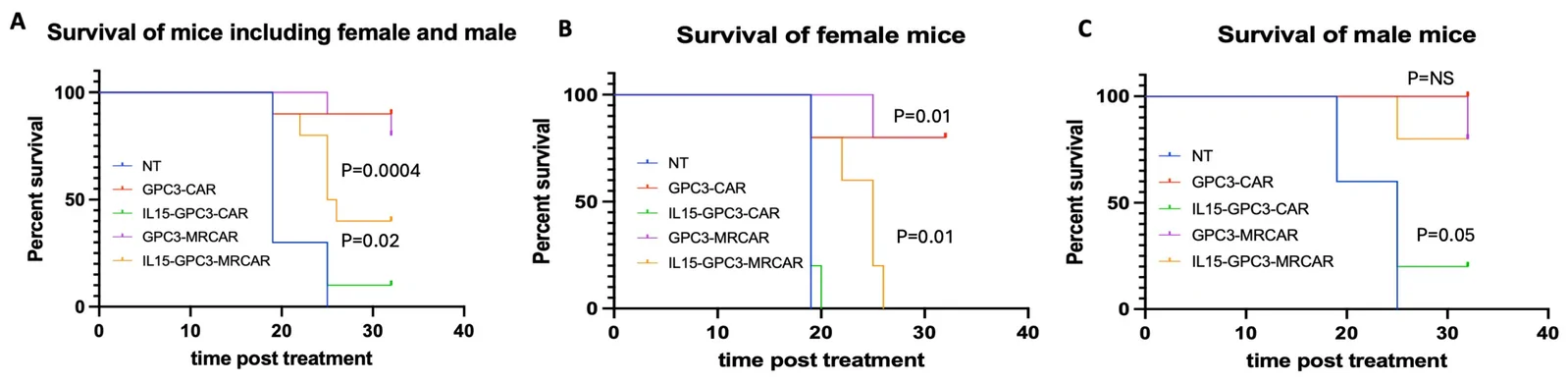

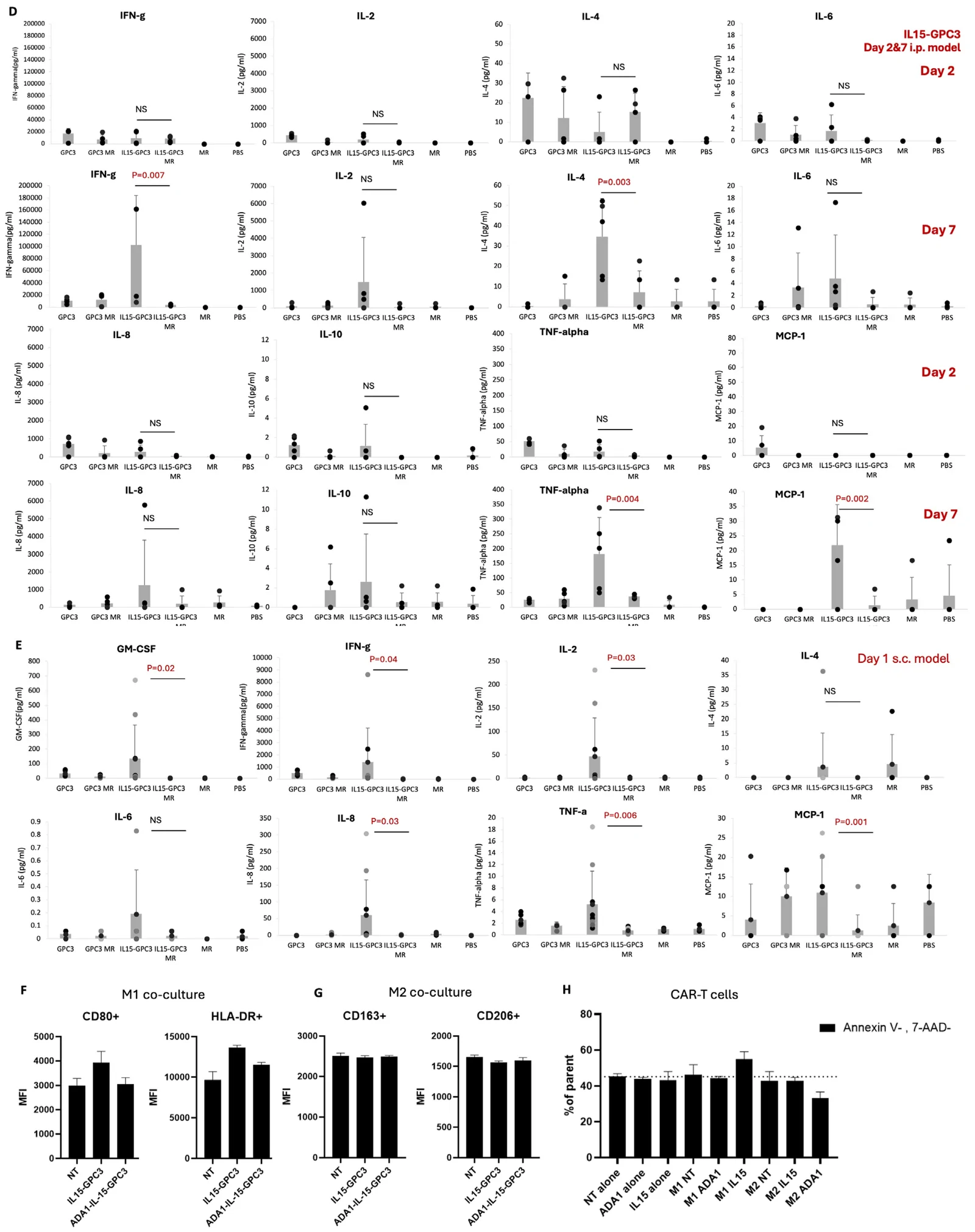
ADA1–CD26 mitigates cytokine release syndrome and improves survival in HCC model. To establish a systemic toxicity model, NSG mice were engrafted intraperitoneally (i.p.) with 2 × 10^6^ Huh7 tumor cells, followed by i.p. administration of 1 × 10^7^ CAR T cells together with 1 × 10^7^ PBMCs. (**A**–**C**) Kaplan–Meier survival curves of female and male NSG mice bearing Huh7 tumors following CAR T-cell treatment (survival data from 5 male and 5 female mice per group; statistical significance was determined using the log-rank Mantel–Cox test comparing the indicated treatment groups). (**D**,**E**) Serum cytokine levels measured in peripheral blood collected on days 2 and 7 after CAR T-cell administration (data are presented as mean ± SEM from *n* = 5–10 mice per group; statistical differences were evaluated using a two-way ANOVA with Tukey’s multiple comparisons test comparing all treatment groups across time points). (**F**–**H**) In vitro macrophage co-culture: quantification of M1-like (**F**) and M2-like (**G**) polarization and cytokine profile after exposure to IL15-GPC3-CAR or MRCAR T cells (data are presented as mean ± SEM from *n* = 3 independent biological experiments; statistical significance was determined using a one-way ANOVA followed by Tukey’s multiple comparisons test comparing all tested groups). (**H**) T cell expansion in macrophage/T cell cocultures with GPC3-Huh7 targets, showing enhanced proliferation and viability in the MRCAR group (data are presented as mean ± SEM from *n* = 3 independent biological experiments; statistical significance was determined using a one-way ANOVA followed by Tukey’s multiple comparisons test comparing all tested groups).

To determine whether incorporation of ADA1 and CD26 could mitigate inflammatory toxicity while preserving antitumor efficacy, NSG mice bearing intraperitoneal (i.p.) luciferase-expressing Huh7 tumors were treated with either MRCAR or conventional CAR T cells (*n* = 5 female and 5 male mice per group). All GPC3-targeted CAR T-cell treatment groups achieved complete tumor clearance by one week after treatment, as evidenced by in vivo bioluminescence imaging. Despite this comparable antitumor response, mice treated with IL15-GPC3-CAR T cells exhibited substantial mortality, with survival declining in a pattern similar to that observed in untreated tumor-bearing mice in both female and male cohorts. In contrast, treatment with IL15-GPC3-MRCAR T cells significantly improved survival in both female and male mice compared with conventional IL15-GPC3-CAR T cells (Figure 9A–C). These findings suggest that incorporation of ADA1 and CD26 substantially improves the safety profile of IL15-armed GPC3-CAR T cells without compromising their antitumor efficacy.

**Figure 10:**
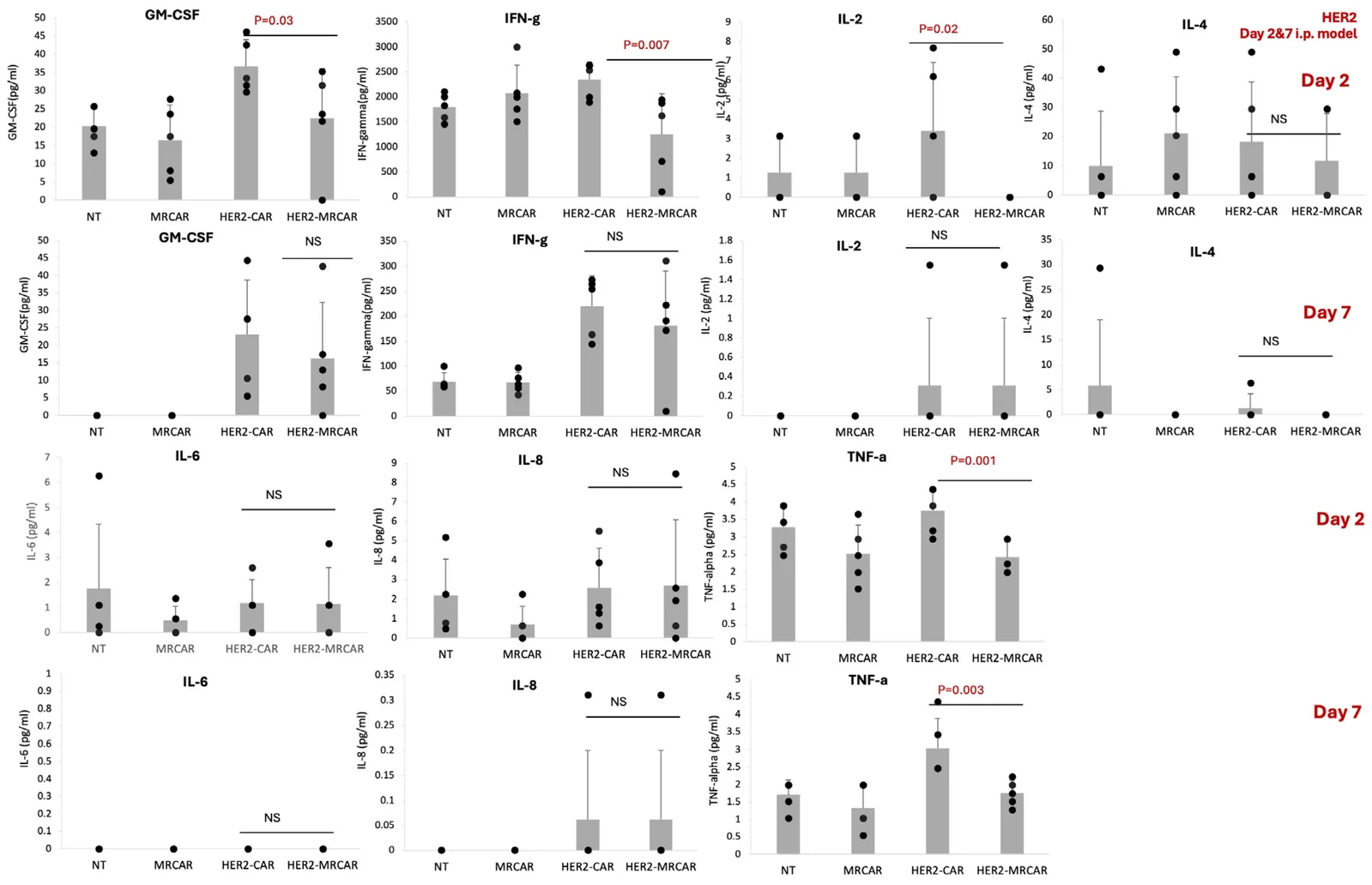
ADA1–CD26 mitigates systemic toxicities in T47D model. NSG mice were engrafted with 1 × 10^7^ T47D tumor cells in the 4th mammary fat pad, followed by i.p. administration of 1 × 10^7^ CAR T cells together with 1 × 10^7^ PBMCs. Serum cytokine levels measured in peripheral blood collected on days 2 and 7 (highlighted in Red) after CAR T-cell administration (data are presented as mean ± SEM, where each individual dot represens data from a single mouse, *n* = 5 mice per group; statistical significance where *p* < 0.05 is highlighted in red was determined using a two-way ANOVA followed by Tukey’s multiple comparisons test comparing all treatment groups across the time points).

Multiplex cytokine analysis further demonstrated that MRCAR treatment attenuated systemic inflammatory responses following CAR T-cell administration. In the i.p. Huh7 model, serum cytokine profiling at days 2 and 7 revealed distinct inflammatory responses between conventional IL15-GPC3-CAR and IL15-GPC3-MRCAR T-cell treatments. At day 2, serum IFN-γ and IL-4 levels were significantly elevated following IL15-GPC3-CAR T-cell treatment, whereas these increases were not observed in the MRCAR-treated group. By day 7, TNF-α and MCP-1 were similarly increased in mice treated with IL15-GPC3-CAR T cells but remained comparatively low following MRCAR treatment (*n* = 3–5 per time point; Figure 9D). A similar pattern of reduced systemic cytokine production following MRCAR treatment was observed in the HER2-targeted CAR T-cell i.p. model at both days 2 and 7 (Figure 10).

Consistent with these findings, in the s.c. Huh7 model, serum levels of GM-CSF, IFN-γ, IL-2, IL-8, TNF-α, and MCP-1 were significantly elevated one day after administration of IL15-GPC3-CAR T cells compared with untreated mice, whereas these cytokines were not significantly increased following IL15-GPC3-MRCAR treatment (Figure 9E). Because CAR T cells administered systemically in the s.c. model are unlikely to have substantially encountered the tumor within this early time window, the acute cytokine response may, at least in part, reflect antigen-independent or nonspecific immune activation associated with IL15-armed CAR T cells rather than tumor-directed activity. Collectively, these findings suggest that conventional IL15-CAR T cells can induce excessive systemic cytokine activation, whereas incorporation of ADA1 and CD26 substantially attenuates this response, potentially contributing to the improved safety and survival observed with MRCAR treatment.

Because macrophages can substantially influence inflammation and T-cell function within the tumor microenvironment, macrophage co-culture experiments were performed to further investigate the immunomodulatory effects of MRCAR T cells. Conventional IL15-expressing CAR T cells induced macrophage activation, as evidenced by increased expression of the M1-associated markers CD80 and HLA-DR, whereas IL15-MRCAR T cells markedly attenuated this inflammatory response (one-way ANOVA with Tukey post-hoc test; IL-15-GPC3 group showed significantly increased CD80 and HLA-DR expression compared to ADA1-IL15-GPC3 group, Figure 9F). Importantly, the M2-associated markers CD163 and CD206 showed no significant change between groups (Figure 9G). Notably, conventional IL15-CAR T cells also exhibited greater expansion in the presence of macrophages (one-way ANOVA with Tukey post-hoc test; M1 IL15 group showed significantly higher expression compared to other groups, Figure 9H), suggesting that this enhanced proliferation may be accompanied by increased macrophage-mediated inflammatory activation. In contrast, MRCAR T cells maintained their therapeutic activity while limiting this proinflammatory macrophage response. Collectively, these findings support a role for ADA1–CD26 rewiring in improving CAR T-cell persistence and antitumor activity while simultaneously attenuating inflammatory activation and promoting a less inflammatory tumor immune microenvironment.

## 4. Discussion

The persistence, expansion, and safe application of CAR T cell therapies in solid tumors remain among the greatest unmet challenges in cancer immunotherapy. Our study demonstrates that metabolic rewiring of CAR T cells via ADA1 and CD26 co-expression, coupled with IL-15 support, profoundly augments their antitumor capabilities while substantially reducing cytokine release syndrome. Our survival analyses in Figure 9A–C (supported by the phenotypic data in Figure 9D–H) demonstrate that the inclusion of IL-15 results in accelerated mortality and shorter host survival. Mechanistically, IL-15 is a potent pro-inflammatory cytokine that heavily promotes the proliferation, hyper-activation, and polarization of M1-like immune cells. While this potent stimulation can enhance effector functions, unmodulated systemic delivery triggers an excessive, toxic inflammatory cytokine cascade that compromises overall host viability. This highlights a critical therapeutic challenge: balancing enhanced activation with systemic safety. Importantly, our ADA1-CD26-based rewiring approach offers a promising framework to regulate and dampen this excessive hyper-inflammation, providing a potential pathway to harness the benefits of cytokine support while mitigating the adverse survival outcomes associated with unmitigated IL-15 signaling.

This multi-receptor CAR approach addresses several key limitations encountered in solid tumor settings, including poor T cell infiltration, rapid exhaustion, limited memory formation, and inflammatory toxicity. Through systematic in vitro and in vivo characterization, we show that ADA1–CD26 multiplexing enhances the expansion, cytotoxicity, and survival of CAR T cells in models of hepatocellular carcinoma and breast cancer. ADA1 overexpression enabled T cells to metabolize adenosine, a prevalent immunosuppressive metabolite in the tumor microenvironment, thereby restoring metabolic fitness and supporting sustained effector function. This aligns with previous studies implicating adenosine metabolism as a regulatory mechanism for immune cell suppression and highlights ADA1 as a practical target for engineering metabolically resilient T cells [14,15,25,26]. The supplementary expression of CD26 further boosted T cell invasiveness, facilitating migration through Matrigel barriers and improving homing to tumor sites via MAPK and PI3K signaling pathways. These results build upon prior work identifying CD26^high^ T subsets as critical for tissue infiltration and functional persistence [14,15,28].

Repeated antigen exposure and chronic stimulation are known to induce exhaustion markers and impair CAR T cell efficacy. Here, ADA1–CD26 MRCAR T cells demonstrated marked resistance to exhaustion, maintaining viability and low levels of inhibitory receptor expression after repeated tumor stimulations. The improved expansion and maintenance of memory subsets—including central memory (Tcm) and stem memory (Tscm) populations—provide mechanistic insight into the observed protection against tumor rechallenge in both HCC and breast cancer models. These findings further support the concept that metabolic rewiring promotes a less differentiated phenotype, helping maintain a pool of functional, long-lasting T cells [10,13,45,46,47,48,49,50,51,52].

In this study, we evaluated the impact of PI3K, MEK1, and p38 MAPK inhibition on the invasive capacity of GPC3- and HER2-targeted MRCAR T cells and found that PI3K inhibition uniformly reduced invasion across both models. Interestingly, MEK1 inhibition (PD98059) preferentially impaired invasion by GPC3-MRCAR T cells, while p38 MAPK inhibition (SB203580) had a greater effect on HER2-MRCAR T cells. These results suggest that downstream signaling following CD26/SDF-1α engagement may be influenced by the CAR costimulatory domain utilized in each construct. Specifically, GPC3-MRCAR T cells were engineered with a 4-1BB (CD137) costimulatory domain, whereas HER2-MRCAR T cells contained a CD28 domain. Previous studies have shown that 4-1BB and CD28 costimulation can distinctly modulate T cell signaling and metabolic programming. 4-1BB signaling is typically associated with enhanced MAPK/ERK pathway activity and mitochondrial function, while CD28 more robustly activates PI3K/AKT and p38 MAPK pathways [53,54,55,56,57,58]. CD26 signaling, especially when combined with chemokine receptor CXCR4 and SDF-1α stimulation, is known to engage both MAPK and PI3K signaling cascades [35,41,42]. Based on our data, we propose that the type of CAR endodomain costimulation impacts the integration of CD26/CXCR4 signaling, leading to differential utilization of MEK1 or p38 MAPK pathways in engineered T cells. The interplay between CAR design and microenvironmental cues therefore critically shapes functional outcomes. Future mechanistic studies will be important to dissect the crosstalk between CAR co-stimulatory domains and CD26/CXCR4 in regulating MAPK and PI3K pathway activation.

Importantly, our data indicate that ADA1–CD26 rewiring mitigates the risk of systemic toxicity without compromising therapeutic efficacy. ADA1–CD26 MRCAR T cells produced significantly lower levels of pro-inflammatory cytokines (IL-6, IFN-γ, TNF-α) in vivo and induced changes in macrophage polarization favoring M1-like over M2-like phenotypes, thereby contributing to a more favorable immune profile. This dual effect—improved antitumor response and reduced systemic toxicity—addresses a major safety concern and is likely attributable to both metabolic normalization and immune reprogramming of the tumor microenvironment. Recent clinical experience with IL-15–enhanced CAR T therapies has underscored the risk of hyperinflammation [20], yet our data show that ADA1–CD26 can provide a safety brake even alongside a potent expansion signal.

The translational implications of our findings are significant. By combining metabolic, migratory, and survival enhancements, ADA1–CD26-based CAR T cells may overcome major bottlenecks hampering solid tumor immunotherapy, potentially increasing response rates and treatment durability, while minimizing toxicity. In particular, the sustained efficacy and rechallenge protection observed here support the development of “off-the-shelf” or repeated dosing strategies. This platform may be adapted to multiple antigen targets across cancer types, as illustrated by performance in both GPC3-positive HCC and HER2-positive breast cancer.

Despite these promising results, several limitations warrant further investigation. First, although our murine models recapitulate key aspects of human solid tumors, differences in the human immune response and tumor architecture may influence clinical translation. Second, long-term follow-up studies are needed to determine whether metabolic rewiring affects T cell persistence and function beyond the timeframes studied here. Third, the potential for off-target effects or metabolic imbalances must be evaluated by tracking ADA1 and CD26 expression in non-tumor tissues, as well as monitoring for secondary toxicity. Another limitation of this study is that the relative contributions of ADA1 activity (enzymatic cleavage of extracellular adenosine/ATP) versus CD26-mediated cleavage of chemokines and peptides to the reduction in M1 polarization and proinflammatory cytokine secretion remain unclear. Future studies utilizing selective inhibition or genetic knockdown of ADA1 or CD26 will be necessary to dissect these individual mechanisms. Additionally, although anti-CD3 scFv anchoring could theoretically influence T cell baseline activation, our prior work demonstrated that secretion of ADA1-hCD3scFv is conditional, requiring CD3 and CD26 stimulation [14], and in the present study, surface-anchoring does not lead to spontaneous TCR signaling or activation in the absence of antigenic stimulation. Nonetheless, we acknowledge this potential effect as a limitation. Finally, future work should explore combinatorial approaches involving checkpoint blockade or novel CAR designs to optimize the metabolic and immune landscape.

In conclusion, our study establishes ADA1–CD26 metabolic rewiring as a potent strategy for next-generation CAR T cell therapies in solid tumors, promoting tumor infiltration, persistence, and immune safety. These results provide a robust foundation for clinical development and lay the groundwork for overcoming longstanding barriers in solid tumor immunotherapy.

## Data Availability

The raw data supporting the conclusions of this article will be made available by the authors on request.
